# Artificial blood vessels-clinical development of TEVG

**DOI:** 10.1007/s10047-025-01508-9

**Published:** 2025-05-26

**Authors:** Manabu Itoh, Keiji Kamohara, Koichi Node, Koichi Nakayama

**Affiliations:** 1 Department of Thoracic and Cardiovascular Surgery, Faculty of Medicine, Saga University, 5-1-1 Nabeshima, Saga, 849-8501 Japan; 2 Department of Cardiovascular Medicine, Faculty of Medicine, Saga University, Saga, Japan; 3 Center for Regenerative Medicine Research, Faculty of Medicine, Saga University, Saga, Japan

**Keywords:** Tissue-engineered vascular grafts, Artificial blood vessel, Clinical development

## Abstract

The market for small-diameter vascular grafts (< 6 mm) used in cardiac and vascular surgery has not yet been fully established, as stable long-term patency has not been achieved. This paper focuses on the clinical development of tissue-engineered vascular grafts (TEVGs), especially those that have progressed to clinical trials, and introduces their current status with historical background and future directions. This review was created based on a translation of the Japanese review first reported in the *Japanese Journal of Artificial Organs* in 2023 (vol. 52, no. 3, pp. 161–166), with some modifications.

## Introduction

Although medical technology is constantly evolving, there are still many challenges that have yet to be overcome in clinical practice. For example, the market for small-diameter vascular grafts with a diameter of less than 6 mm used in cardiovascular surgery has not been fully established, because stable long-term patency has not been achieved. Therefore, the development of tissue-engineered vascular grafts (TEVGs) using tissue engineering (TE) technology, which represents a novel approach from traditional synthetic resin products, is attracting attention and expected to address unmet needs.

This paper presents the current status with historical background and offers insights into the future directions, focusing on the clinical development of TEVG, especially those that have progressed to clinical trials.

## History and current status of artificial blood vessel development

The history of artificial blood vessels dates back to the 1950s. In 1952, Voorhees et al. reported the creation of an artificial blood vessel made of Vinyon N (polyvinyl chloride fiber), which at the time was the material used in military parachutes [1]. In 1954, DeBakey used a polyester fiber graft for the replacement of an abdominal aortic aneurysms [2]. Polyester vascular grafts were later branched into woven and knitted forms, and further developed into shield grafts coated with gelatin or collagen to prevent blood leakage through the gaps between the fibers. In addition to the strength of the material itself, polyester vascular grafts have achieved excellent hemostasis and long-term durability due to the pseudo-intimal and pseudo-adventitial formation (organogenesis) resulting from the tissue reaction that occurs when the body tries to somehow accept foreign artificial materials. Currently, polyester artificial blood vessels are the mainstream of large-diameter (inner diameter of 10 mm or more) prosthetic blood vessels used in thoracic and abdominal aortic surgery, and are also widely used as the base material for catheter-based aortic stent grafts, which are widely used as a minimally invasive treatment.

Since the 1970s, expanded polytetrafluoroethylene (ePTFE) has appeared. It is stretched polytetrafluoroethylene (PTFE) to create a porous structure, which is one of the so-called antithrombogenic materials with high hydrophobicity that prevents adhesion of proteins, platelets, and cells. Over the subsequent decades of improvements in layer construction and coating technology, ePTFE grafts are now commonly used as medium-diameter (inner diameter 6 mm or more but less than 10 mm) and small-diameter (inner diameter less than 6 mm) grafts [3]. They have become the mainstay of artificial vessels for arterial bypass surgery in the cervical, abdominal branches and lower extremities, as well as for short-circuit surgery in pediatric congenital heart disease and vascular access reconstruction (arteriovenous shunt) for hemodialysis.

Currently, some artificial blood vessels for dialysis are made of polyurethane resin for early puncture. However, in practical medical application, the majority of artificial blood vessels are composed of polyester fibers and ePTFE. Although the long-term patency and durability of large- and medium-diameter vascular grafts are satisfactory, there are still issues such as decreased patency associated with downsizing. In fact, commercially available small-diameter artificial vessels made of ePTFE differ significantly from biological vessels by having low vascular compliance, one measure of vascular elasticity. Therefore, stress concentration and hydrodynamic turbulence due to non-compliance occur at the anastomosis between the native and artificial blood vessels, leading to thrombus formation in the early stage of implantation and to occlusion due to stenosis caused by intimal thickening at the anastomosis site in the long term [4–6].

How about using one’s own small-caliber substitute blood vessels (autologous vascular grafts)? For example, the internal thoracic artery graft, an elastic artery with a diameter less than 3 mm used for coronary artery bypass grafting, is composed of endothelial cells (ECs), smooth muscle cells (SMCs), and abundant elastic fibers (including internal and external elastic lamina). It exhibits extremely excellent long-term patency, durability, growth potential [7], and self-repair capabilities [8], contributing to improvements in life prognosis [9, 10]. This is considered to be due to the fact that after transplantation into a living body, it integrates with its own tissue (coronary artery) while maintaining moderate flexibility, thereby demonstrating its inherent physiological functions such as contraction/relaxation and antithrombotic properties by regulating blood coagulation and inflammation, and preventing thrombus formation and intimal thickening that can cause blockages [11].

On the other hand, in addition to the issues of thrombus occlusion and intimal hyperplasia mentioned earlier, the artificial blood vessel for dialysis poses the issue of infection from the puncture site [12]. Moreover, not only dialysis artificial blood vessels but also existing artificial vessels made from foreign materials such as synthetic resin, biofilms (bacterial nests) are easily formed when bacteria adhere to the surface of artificial vessels, and if the infection worsens, it may progress to fatal sepsis [13, 14]. Thus, satisfactory small-caliber artificial blood vessels in clinical practice do not exist at present. In order to overcome these challenges, research and development of TEVG have been actively pursued. 

## Clinical development of TEVG

In 1993, Langer et al. proposed the concept of TE [15]. Tissues inherently consist of cells forming aggregates, and the gaps between them are filled with proteins called extracellular matrix (ECM). TE artificially creates such a structure, and it has been common to use biologically derived materials such as collagen or biodegradable high molecular weight polymers like polylactic acid, which is included in medical devices and pharmaceuticals approved for clinical use, as scaffold materials to support cell growth. However, scaffolds, which are essentially foreign to living organisms, cause inflammation as well as a risk of infection that cannot be completely avoided. Therefore, attempts have been made to construct three-dimensional tissues without using scaffolds [16–20].

The manufacturing methods of TEVG can be broadly classified into the scaffold-based method and the scaffold-free method which is based on the ECM produced by their own cells. However, the classification has become more complex due to the diversification of cell sources resulting from recent advances in regenerative medicine technology, the introduction of various scaffold-based induction methods and 3D printing technology, and the combination of these technologies.

Human induced pluripotent stem (iPS) cells represent a groundbreaking achievement in stem cell biology pioneered by Yamanaka and his colleagues, who received the Nobel Prize in 2012. Due to their ability to differentiate into any type of cell, iPS cells hold great potential in personalized medicine [21]. In particular, they have garnered attention for their ability to be derived from easily accessible tissues, such as skin and blood, which allows for avoidance of ethical concerns and reduces immunogenic risks, making them promising for applications in TEVG. However, challenges remain, including the potential for tumor formation, mitochondrial DNA mutations, the complexity of cell reprogramming, and the high cost of the process, all of which present barriers to clinical application. Therefore, to achieve broader clinical use of iPS cells, improvements in safety, cost reduction, and manufacturing process are urgently needed [22–24].

Focusing on TEVG that has reached the stage of human clinical trials, the following sections describe the methods and progress of clinical development of TEVG, although the information is limited to paper reports and publicly available information from domestic and foreign government agencies.

1) Method using biodegradable polymers (scaffold based ①)

In 2001, Shin'oka et al. achieved the first successful clinical application of biodegradable polymers in the field of pediatric cardiac surgery, where expectations for growth were high [25]. TEVG, in which cells from autologous peripheral veins were seeded into biodegradable polymers, was used as an epicardial conduit during the Fontan procedure to connect the inferior vena cava to the pulmonary artery. In a follow-up study of 25 patients transplanted between 2001 and 2004, no graft infection, varicose veins, or calcification was found, with the most frequent problem being graft stenosis. Overall, 24% of patients required balloon angioplasty for asymptomatic graft stenosis [26]. Subsequently, basic research on TEVG using various types and shapes of biodegradable polymers has progressed with the aim of adjusting mechanical properties and degradation rates. Currently, a phase II clinical trial led by physicians using second-generation TEVG is underway [National Clinical Trials Network (NCTN) (NCT04467671)]. In this trial, TEVG was prepared by aseptically seeding high-density autologous bone marrow cells onto biodegradable polymers using a special closed circuit in the operating room. The objectives of the study include evaluating the incidence of complications such as stenosis, thrombus formation, infection, and rupture, as well as examining the growth and remodeling mechanisms of the TEVG [27]. In this study, asymptomatic stenosis was observed in three of the four patients implanted as epicardial conduits in the Fontan procedure, and balloon angioplasty was performed. However, computational fluid dynamics (CFD) simulations showed that, despite the stenosis, the hemodynamic parameters remained within acceptable limits. The TEVGs in this trial are conditioned to have diameters ranging from 12 to 24 mm. In addition, because the Fontan circulation is characterized by high-volume flow and low-pressure conditions, the risk of fatal complications such as acute thrombosis, dilation, or rupture of the TEVG is relatively low. Therefore, this approach limits the implantation site to the low-pressure, high-flow, large-caliber venous system (right heart system).

A recent focus of attention in the small-diameter vascular prostheses is Xeltis’ TEVG, which uses a biodegradable polymer based on polycarbonate urethane (PCU). As a dialysis prosthesis with a diameter of 6 mm, a first-in-human trial (NCT04898153) involving 20 patients with end-stage renal failure reported favorable results with a primary patency rate of 80% and a secondary patency rate of 100% over a 6-month period. Although some cases required reintervention, no infections were reported [28]. In addition, a pivotal trial (NCT05473299) involving 110 patients with end-stage renal failure is designed to evaluate the safety and performance of the dialysis access graft (aXess). Currently, 21 institutions are enrolled in this trial, but the results, including interim reports, have not yet been published.

More notably, a first-in-human clinical trial (NCT04545112) using a 4 mm diameter coronary artery bypass graft (XABG), which has been long awaited worldwide, is currently underway. This trial aims to evaluate the safety and efficacy of XABG in adult patients who are eligible for coronary artery bypass graft surgery due to coronary artery disease. Specifically, this trial aims to assess the incidence of postoperative complications (e.g., infection, thrombus formation, graft occlusion) associated with the use of XABG, to track blood flow and graft patency over a certain period. Additionally, this study also examines the impact on patients' cardiac function and postoperative quality of life (QOL) by comparing the grafts to great saphenous vein grafts (SVG) and internal thoracic artery grafts, which are commonly used in clinical practice.

An animal study was conducted in sheep as a preclinical study of XABG. The 1-year patency rate of XABG grafts (4 mm in diameter, 15 cm in length) used for coronary artery bypass in sheep was 72.7% (8 of 11 grafts). In contrast, the control group, which used saphenous vein grafts (SVG), showed dilatation, while XABG demonstrated excellent blood flow and uniformity in diameter. These results confirm that XABG exhibited the longest patency as a coronary artery bypass graft in a large animal studied to date [29].

2) Method using decellularized ECM (scaffold based ②)

ECM, which is obtained by removing antigenic components such as cells from human, bovine or porcine tissues, and organs, has been attracting attention as a scaffold that more closely reproduces living organisms. The current goal of decellularization is minimizing the destruction of the ECM while maximizing the efficiency of cellular component removal. There are three main methods: chemical, biological, and physical [30]. Several small-diameter decellularized grafts were commercially available, pioneered by bovine carotid artery [Artegraft^®^ (LeMaitre Vascular)], bovine ureter [SynerGraft^®^Vascular Graft Model 100 (CryoLife)], and human saphenous vein [Cryovein^®^ (Artivion)]. Artegraft^®^ was approved in the United States and Europe in 1970 and has been implanted in more than 500,000 patients over the past 50 years as an artificial blood vessel for dialysis and lower extremity arterial bypass [31].

Many studies, including prospective randomized trials, have shown that the patency rates of these decellularized heterogeneous grafts are comparable to those of synthetic grafts such as ePTFE, with no clear superiority demonstrated [32, 33]. In addition, decellularized heterogeneous grafts are less likely to be salvaged in the event of complications such as infection or pseudoaneurysm compared to synthetic grafts, and are also more expensive [34].

In addition, a small-diameter vascular graft made from a sheet of decellularized porcine aortic tunica media, which was then rolled into a graft, has been reported, although it is still in the basic research stage. This graft was implanted in the carotid artery of rats and was confirmed to maintain patency while exhibiting antithrombotic properties [35]. An animal transplantation study using small-diameter decellularized grafts (2 mm inner diameter, 20–30 cm in length) made from ostrich carotid arteries with peptides arranged on the luminal surface to supplement CD34-positive endothelial cells has also been reported. This graft was implanted as a femoral-to-femoral artery bypass in miniature pigs and showed good patency after 20 days of observation without the use of anticoagulants [36].

3) Combination method using biodegradable polymer and decellularized ECM (scaffold based ③)

Method using biodegradable polymers and decellularized ECM include the Humacyte human acellular vessel (HAV) by Niklason et al. When first developed in 1999, it was a living TEVG that incorporated SMC and EC into a biodegradable polymer [37]. The primary source of SMCs and ECs is the patient themselves, also referred to as autologous vascular cells. The advantage of this source lies in its immunocompatibility; however, several drawbacks exist. First, these cells are usually collected by vascular biopsy, a procedure that is invasive and carries the risk of complications at the donor site. Second, due to the old age of the donor and the primary nature of the cells, they have low proliferative and regenerative capacity, limiting the quantity of available cells [23]. Therefore, there were limitations in the collection of SMC for clinical application, and a switch was made to the use of allogeneic donor cells. Specifically, SMCs derived from the aorta of an organ donor (allogeneic donor) are seeded onto biodegradable polymers and cultured under pulsatile circulation in a bioreactor. In this process, the polymers are almost completely degraded to form TEVG, which is composed mainly of collagen produced by SMC. After eight weeks of culture under pulsatile circulation, the resulting TEVG is decellularized and the homologous antigen is removed. The resulting small-caliber TEVGs were implanted as dialysis vessels in 60 patients with end-stage renal disease in the United States and Poland in 2012. No immune rejection was observed during post-transplant follow-up, and the primary patency rate in a Phase II clinical trials (NCT01744418, NCT01840956) were 63% at 6 months and 28% at 1 year after transplantation [38]. No advantage over ePTFE was demonstrated at this time point. Histological analysis of grafts obtained from eight patients at 16–55 weeks post-transplantation showed less inflammation and good autologous tissue invasion consisting of endothelialization and vascular SMCs on the luminal side of graft wall [38], followed by a good secondary patency rate of 58.2% at 5 years (39.2–73.1 months) post-transplantation [39]. Two phase III clinical trials comparing HAV, ePTFE, and the acellular tissue-engineered vessel (ATEV) with autologous arteriovenous shunts are currently ongoing (NCT02644941, NCT03183245). In a phase III clinical trial comparing HAV and ePTFE for hemodialysis access (NCT02644941), it was demonstrated that the infection rate of HAV (*n* = 177) was significantly lower than that of ePTFE (*n* = 178) (0.93% vs 4.54%, *P* = 0.0413) [40]. Regarding NCT03183245, which compares ATEV and autologous arteriovenous shunts, the trial results were reported in a press release on July 31, 2024 (https://investors.humacyte.com/news-releases/news-release-details/humacyte-acellular-tissue-engineered-vessel-atevtm-meets-primary/) and a subsequent press release on October 8, 2024 (https://investors.humacyte.com/news-releases/news-release-details/humacyte-late-breaking-abstract-accepted-oral-presentation-v007). NCT03183245 was a prospective, multicenter, randomized clinical trial involving 242 hemodialysis patients, who were randomly assigned to receive either ATEV or autologous arteriovenous shunts for hemodialysis access, with follow-up up to 24 months. ATEV demonstrated superiority over autologous arteriovenous shunts in terms of functional patency (at 6 months: 81.3% vs. 66.4%, at 12 months: 68.3% vs. 62.2%, *P* = 0.0071). The results of this trial were presented orally at Kidney Week of the American Society of Nephrology on October 26, 2024(https://www.asn-online.org/education/kidneyweek/2024/program-abstract.aspx?controlId=4176824).

Moreover, HAV was evaluated in a phase II clinical trial (NCT01872208) as a graft for lower extremity artery bypass (from the femoral artery to the above-knee popliteal artery) in patients with peripheral arterial disease, with results of 20 transplanted cases reported. The primary and secondary patency rates at 2 years post-implantation were 58% and 74%, respectively, falling within the range of patency rates for ePTFE and autologous vein grafts. Additionally, no limb amputations or graft infections were observed in the TEVG-implanted limbs during the 2-year follow-up [41]. Furthermore, phase II clinical trials (NCT03005418, NCT05873959) involving patients with extremity vascular injuries due to war or traffic trauma suggested superiority over existing synthetic grafts in terms of patency, limb preservation, and infection resistance at 30 days post-surgery [42]. According to a press release on December 19, 2024 (https://investors.humacyte.com/news-releases/news-release-details/humacyte-announces-fda-approval-symvesstm-acellular-tissue), the U.S. Food and Drug Administration (FDA) has approved the use of acellular tissue-engineered vessel-tyod as a vascular conduit in adults with extremity arterial injuries when urgent vascular reconstruction is needed to avoid imminent limb loss or when autologous vein graft is not feasible.

4) Cell sheet fabrication method (scaffold free ①)

Tissue-Engineered Blood Vessel (TEBV) is a cell sheet fabrication method by L'Heureux et al. Autologous skin-derived fibroblast (FC) sheets prepared in monolayer culture were wrapped around a cardiac rod to form a multilayer and aged under pulsatile circulating culture in a bioreactor. The cardiac rod was removed, and finally, the artificial vessel was seeded on the luminal surface with autologous vascular EC harvested from a superficial vein [43]. Because it does not use any exogenous scaffolds derived from animal or synthetic components, this approach has attracted attention for its potential to completely avoid issues related to foreign body infection and biotoxicity. The graft, named Lifeline, underwent a phase II clinical trial as a dialysis vascular graft from 2004 to 2007. Among the 10 enrolled dialysis patients, one patient dropped out of the study just before transplantation due to severe gastrointestinal bleeding, and another patient died of an unrelated cause with an open graft during the observation period. Of the remaining 8 patients, 5 (60%) maintained primary patency at 6 months, while the other 3 experienced dysfunction, which was attributed to thrombus occlusion and mass formation [44]. The subsequent 12-month primary and secondary patency rates were 86%, and the longest primary patency period was reported to be 38.6 months, indicating favorable long-term remote outcomes [45]. However, the production of the graft in vitro required a long period of 6–9 months, which posed challenges in terms of patient waiting period and high cost, making it unfeasible as an industrial product [46].

In the next second-generation model, a tubular tissue was developed not from autologous but from allogeneic FCs and was improved as an “off-the-shelf graft” that can be freeze-preserved after undergoing a drying process. The seeding of EC onto the luminal surface was also omitted. This graft was allogeneically transplanted into three patients in a clinical trial. Since FC in culture does not express major histocompatibility complex class II antigens, decellularization prior to transplantation was not considered essential when using FC alone. Therefore, deactivation method involving drying and freezing was employed rather than decellularization to reduce the antigenicity associated with allogeneic transplantation. No adverse immune reactions were observed in all three transplanted patients [47]. One patient lost graft patency 11 months after transplantation due to an automobile accident; one patient developed septic 6 weeks after transplantation and the Lifeline graft was the only vascular access for the last 7 days before death. Autopsy revealed multiple renal abscesses with lung abscesses; one patient experienced thrombotic occlusion 7 months after transplantation. As with the first-generation model, industrialization was not achieved [46, 47].

In both the first- and second-generation models described above, cell assembled membrane (CAM) sheets were successfully rolled to form tubes and used as TEVG [43, 44, 48]. However, as with many tissue engineering approaches, they were complex, required specially designed equipment, and could only be manufactured in a limited number of specific structures [49]. The third-generation model was developed to overcome these challenges and improve industrial scalability and productivity. After drying homologous FC sheets called CAM, they were cut into thin strips and twisted to form fibers, which were then braided together to form tubes. The same deactivation method as described above resulted in less inflammatory reactions after implantation and demonstrated sufficient mechanical strength and increased productivity. Mechanical strength under rupture pressure has been reported to exceed that of the human internal thoracic artery. To assess the feasibility of the grafting operation, an experimental graft to a sheep carotid artery (*n* = 1) was performed, but long-term patency has not been evaluated [49, 50]. The clinical trial has not been published at this time, but only the basic research phase has been reported.

5) Method of forming in the body by in-body tissue architecture (scaffold free ②)

The in-body tissue architecture is a technique in which a mold made of silicon or other base material is implanted under the skin of a living body to produce collagen-based tissue produced by FCs using a bio-defense reaction. This technique was first attempted by Sparks et al. after 1968, and clinical trials were conducted using polyester fibers as reinforcement material. However, issues such as thrombus formation and aneurysms occurred after implantation, and this artificial blood vessel, called Sparks' mandril graft, was not widely used [31, 51, 52].

Subsequently, the “biotube” as a small-diameter TEVG was reported in 2004 [53], and research and development of devices and tissue processing methods for in-body tissue architecture have advanced. The biotube was primarily treated with ethanol prior to implantation, and a first-in-human clinical trial was conducted as a dialysis vascular graft. No thrombus occlusion or aneurysm was observed, but two of the two patients had stenosis of the biotube within 4 months after implantation. One patient required frequent Percutaneous Transluminal Angioplasty (PTA) for the stenotic area, which was subsequently replaced with a PTFE graft 30 months after implantation and later occluded [54]. A clinical trial was also conducted in pediatric cardiac surgery as a vascular wall patch for pulmonary arterioplasty, and no stenosis or aneurysm formation was observed in the patch graft at 9 months after implantation [55].

In a first-in-human clinical trial for artificial blood vessel for lower extremity arterial bypass, a distal bypass below the knee was performed by connecting a biotube to an ePTFE graft in patients with severe limb ischemia who could not use an autologous vein graft. Three months after surgery, the ischemic leg wound had completely healed, and an improvement in intermittent claudication was observed. Ultrasonography at 1 year postoperatively confirmed the patency of the graft and showed no thrombus or stenosis [56]. A phase II clinical trial has been underway since 2022 for use as lower extremity artery bypass (above knee level) grafts in patients with critical lower extremity ischemia [Japan Registry of Clinical Trials (jRCT) (jRCT2072220062)]. The results of the trial have not been published at this time.

Although still in the basic research stage, attempts are underway to decellularize allogeneic and xenogeneic biotubes for off-the-shelf grafts beyond the framework of autografts. As an allogeneic transplant, a biotube (5 mm in diameter) produced subcutaneously in a beagle dog was decellularized and implanted into the common carotid artery of a dog of the same breed as a vascular patch. Patency was confirmed without any stenosis or aneurysm formation at 1 month after implantation [57]. As a xenogeneic transplantation, a biotube (2 mm in diameter) produced subcutaneously in a beagle dog was decellularized and implanted into the abdominal aorta of a rat. Patency was confirmed without any stenosis or aneurysm formation 1 month after implantation [58].

6) Method using three-dimensional bioprinting technology (scaffold free ③)

**Fig. 1:**
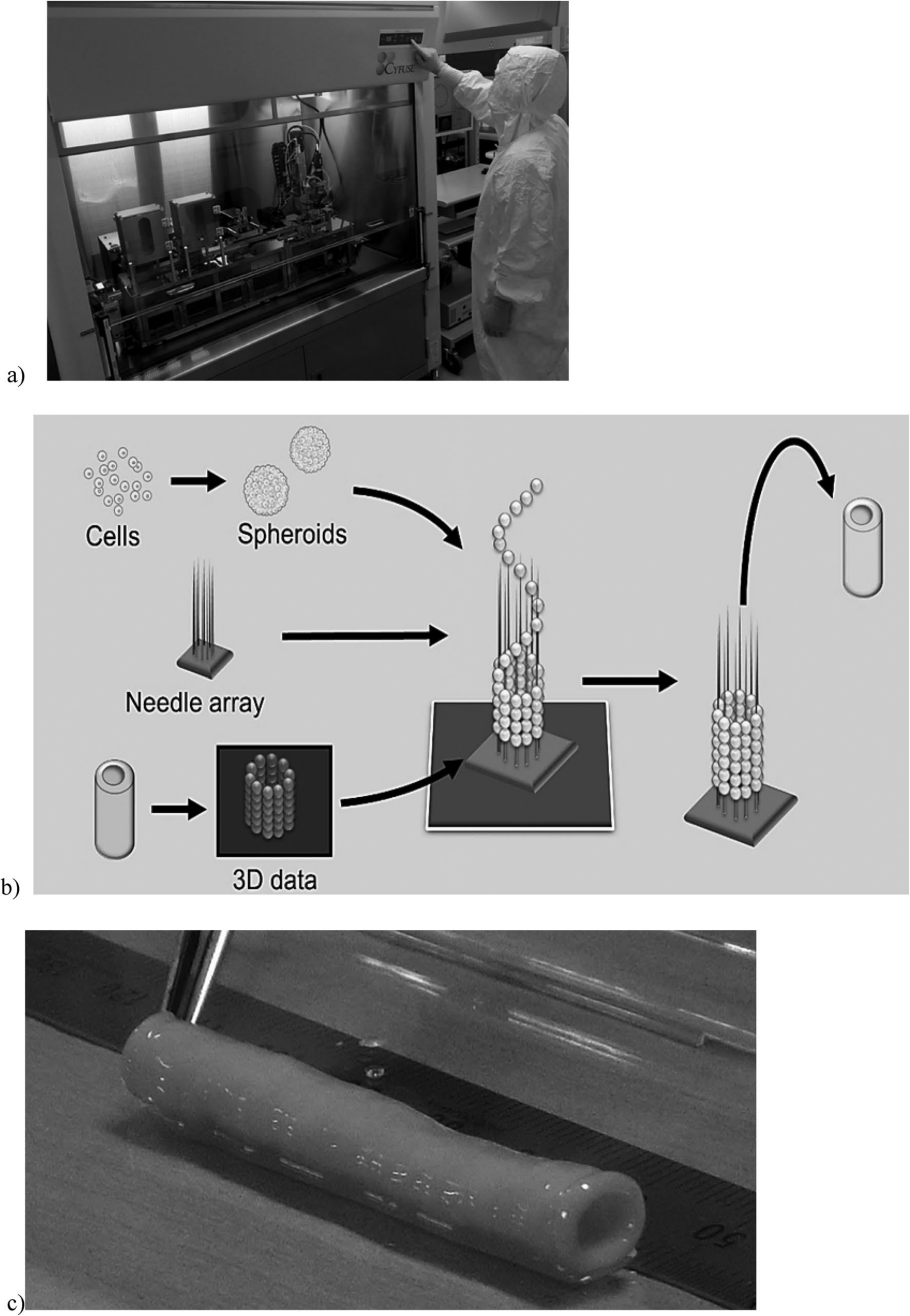
Kenzan Method Bio 3D Printer. **a** Bio 3D printer for clinical use installed in a clean room (jointly developed by Shibuya Corporation and Cyfuse Biomedical K.K.), **b** schematic diagram of the printing method of the bio 3D printer, **c** artificial blood vessel made of small diameter cells consisting only of patient skin-derived fibroblasts and ECM secreted by the cells. Courtesy of Cyfuse Biomedical K.K

Three-dimensional (3D) bioprinting technology, which uses cells to create three-dimensional structures, has been advancing rapidly. In the well-known printing method, a hydrogel such as matrigel or collagen mixed with cells is used as a scaffold to support the cells [59, 60]. On the other hand, Nakayama et al. developed a printing technology that can produce 3D tissue without using any external scaffolds [16, 61]. It is the Kenzan Method Bio 3D Printer (Fig. 1). The main feature of this methods is that spheroids, which are formed by the self-assembling ability of cells, are temporarily fixed with a "Kenzan" to create a 3D shape by fusing the spheroids. The spheroids arranged according to the 3D data fuse with each other, and the Kenzan is finally removed, resulting in a 3D structure consisting only of cells and cell-secreting ECM.

In a preclinical study using this technology, spheroids formed by aggregation of human dermal fibroblasts were used to construct scaffold-free tubular tissue with a diameter of 5 mm using a bio 3D printer. This tubular tissue was transplanted as a cervical arteriovenous shunt in immunodeficient pigs, and stable patency for 3 months was confirmed [62]. Furthermore, a clinical trial is underway for autologous transplantation of the artificial blood vessel for reconstruction of vascular access in dialysis patients (jRCTb070190033). The target populations are hemodialysis patients who are considered to require stenosis replacement, bypass, or aneurysm replacement. Spheroids are made from fibroblasts isolated from skin tissue, and scaffold-free autologous cell-based artificial blood vessels produced using the Kenzan Method Bio 3D Printer are implanted [63]. The results have not been published at this time.

## Conclusion

Artificial blood vessels made of synthetic resins have a history of 70 years, and the history of TEVG since the concept of TE was proposed is less than half of that. In writing this paper, we were able to review the efforts and valuable results of those who pioneered the development of TEVG. The development of TEVG for practical use is accelerating due to the next generation and integration of various TE technologies in Japan and abroad, as well as the addition of new technological innovations. Until the early 2000s, the development of TEVG was aimed at creating grafts in vitro that reproduced the cellular composition, vessel wall structure, and strength similar to those of living blood vessels [35, 64–66]. In recent years, however, there has been a shift in approach to designing products with an eye toward the transplanted TEVG maturing in vivo and becoming a "living vessel." The transplanted TEVG utilizes the biological environment as a bioreactor and acquires vascular wall structure, strength, and physiological function over a long period of time after transplantation, becoming a highly functional vessel comparable to a native vessel.

While the scope of TEVG clinical trials has been mostly limited to pediatric cardiovascular surgery and dialysis, it is only recent that the scope been expanded to include the long-awaited arterial bypass grafts with a diameter of 4 mm or less, distal bypass below the knee, and, finally, coronary artery bypass, which is the ultimate goal. In addition, with a view to practice use and commercialization, fundamental research has been actively conducted in recent years toward ready-made products by improving productivity and functionality through the use of allogeneic cells, including universal iPS cells (induced pluripotent stem cells) that do not carry specific human leukocyte antigens, and by the development of preservation methods [67, 68]. Further development of TEVG is expected in future. 

## References

[1] Blakemore AH, Voorhees AB Jr. The use of tubes constructed from vinyon “N” cloth in bridging arterial defects-experimental and Clinical. Ann Surg. 1954;135(140):324–33. 10.1097/00000658-195409000-00008 PMC1609759 13198069

[2] De Bakey ME. Successful resection of aneurysm of distal aortic arch and replacement by graft. J Am Med Assoc. 1954;155:1398–403. 13183754 10.1001/jama.1954.03690340020007

[3] Piffaretti G, Dorigo W, Castelli P, et al. Results from a multicenter registry of heparin-bonded expanded polytetrafluoroethylene graft for above-the-knee femoropopliteal bypass. J Vasc Surg. 2018;67:1463-71.e1. 29175037 10.1016/j.jvs.2017.09.017

[4] Hayashi H. Artificial blood vessels and rheology. J Jpn Soc Biorheol (B&R). 1990;4:69–80 (**Written in Japanese**).

[5] Abbott WM, Megerman J, Hasson JE, et al. Effect of compliance mismatch on vascular graft patency. J Vasc Surg. 1987;5:376–82. 3102762

[6] Yao Y, Pohan G, Cutiongco MFA, et al. In vivo evaluation of compliance mismatch on intimal hyperplasia formation in small diameter vascular grafts. Biomater Sci. 2023;11:3297–307. 36943136 10.1039/d3bm00167a PMC10160004

[7] Kitamura S, Seki T, Kawata K, et al. Excellent patency and growth potential of internal mammary artery grafts in pediatric coronary artery bypass. New evidence for a “live” conduit. Circulation. 1988;78:I129–39. 3261649

[8] Forouzandeh F, Douglas JS Jr. The left internal mammary artery graft: durable and self-reparative. JACC Case Rep. 2019;1:168–70. 34316777 10.1016/j.jaccas.2019.05.033 PMC8301500

[9] Cameron A, Davis KB, Green G, et al. Coronary bypass surgery with internal-thoracic-artery grafts—effects on survival over a 15-year period. N Engl J Med. 1996;334:216–20. 8531997 10.1056/NEJM199601253340402

[10] Raza S, Blackstone EH, Houghtaling PL, et al. Influence of diabetes on long-term coronary artery bypass graft patency. J Am Coll Cardiol. 2017;70:515–24. 28750693 10.1016/j.jacc.2017.05.061

[11] Kitamura S. Coronary artery bypass surgery and clinical vascular physiology. J Jpn Coll Angiol. 2010;50:247–55 (**Written in Japanese**).

[12] Nassar GM, Ayus JC. Infectious complications of the hemodialysis access. Kidney Int. 2001;60:1–13. 10.1046/j.1523-1755.2001.00765.x. 11422731 10.1046/j.1523-1755.2001.00765.x

[13] Bergamini TM, Bandyk DF, Govostis D, et al. Infection of vascular prostheses caused by bacterial biofilms. J Vasc Surg. 1988;7:21–30. 2961893

[14] Revest M, Camou F, Senneville E, et al. Medical treatment of prosthetic vascular graft infections: review of the literature and proposals of a Working Group. Int J Antimicrob Agents. 2015;46:254–65. 10.1016/j.ijantimicag.2015.04.014. (**Epub 2015 Jum 6**). 26163735 10.1016/j.ijantimicag.2015.04.014

[15] Langer R, Vacanti JP. Tissue engineering. Science. 1993;260:920–6. 8493529 10.1126/science.8493529

[16] Nakayama K. In vitro biofabrication of tissue and organs. Biofabrication. 2013. 10.1016/B978-1-4557-2852-7.00001-9.

[17] Morita S, Noguchi R, Noide T, et al. Recent advances in artificial organ research by regenerative medicine: construction of scaffold-free heart and vascular tissue. Artif Organs. 2012;41:168–71 (**Written in Japanese**).

[18] Sawa Y, Miyagawa S, Sakaguchi T, et al. Tissue engineered myoblast sheets improved cardiac function sufficiently to discontinue LVAS in a patient with DCM: report of a case. Surg Today. 2012;42:181–4. 22200756 10.1007/s00595-011-0106-4

[19] Shimizu T, Yamato M, Kikuchi A, et al. Two-dimensional manipulation of cardiac myocyte sheets utilizing temperature-responsive culture dishes augments the pulsatile amplitude. Tissue Eng. 2001;7:141–51. 11304450 10.1089/107632701300062732

[20] Miyagawa S, Sawa Y, Sakakida S, et al. Tissue cardiomyoplasty using bioengineered contractile cardiomyocyte sheets to repair damaged myocardium: their integration with recipient myocardium. Transplantation. 2005;80:1586–95. 16371930 10.1097/01.tp.0000181163.69108.dd

[21] Takahashi K, Tanabe K, Ohnuki M, et al. Induction of pluripotent stem cells from adult human fibroblasts by defined factors. Cell. 2007;131:861–72. 10.1016/j.cell.2007.11.019. 18035408 10.1016/j.cell.2007.11.019

[22] Goushki MA, Kharat Z, Kehtari M, et al. Applications of extraembryonic tissue-derived cells in vascular tissue regeneration. Stem Cell Res Ther. 2024;15:205. 10.1186/s13287-024-03784-3. 38982541 10.1186/s13287-024-03784-3 PMC11234723

[23] Jouda H, Larrea Murillo L, Wang T. Current progress in vascular engineering and its clinical applications. Cells. 2022. 10.3390/cells11030493. 35159302 10.3390/cells11030493 PMC8834640

[24] Generali M, Casanova EA, Kehl D, et al. Autologous endothelialized small-caliber vascular grafts engineered from blood-derived induced pluripotent stem cells. Acta Biomater. 2019;97:333–43. 10.1016/j.actbio.2019.07.032. (**Epub 2019 Jul 22**). 31344511 10.1016/j.actbio.2019.07.032

[25] Shin’oka T, Imai Y, Ikada Y. Transplantation of a tissue-engineered pulmonary artery. N Engl J Med. 2001;344:532–3. 11221621 10.1056/NEJM200102153440717

[26] Hibino N, McGillicuddy E, Matsumura G, et al. Late-term results of tissue-engineered vascular grafts in humans. J Thorac Cardiovasc Surg. 2010;139(431–6):436.e1-2. 10.1016/j.jtcvs.2009.09.057 20106404

[27] Schwarz EL, Kelly JM, Blum KM, et al. Hemodynamic performance of tissue-engineered vascular grafts in Fontan patients. NPJ Regen Med. 2021;6:38. 34294733 10.1038/s41536-021-00148-w PMC8298568

[28] Tozzi M, De Letter J, Krievins D, et al. First-in-human feasibility study of the aXess graft (aXess-FIH): 6-month results. J Vasc Access. 2024. 10.1177/11297298231220967. (**Online ahead of print**). 38317272 10.1177/11297298231220967

[29] Ono M, Kageyama S, O’Leary N, et al. 1-year patency of biorestorative polymeric coronary artery bypass grafts in an ovine model. JACC Basic Transl Sci. 2023;8:19–34. 36777172 10.1016/j.jacbts.2022.06.021 PMC9911320

[30] Kishida A. Current status and future prospects of decellularized biological tissues. Organ Biol. 2018;25:27–34 (**Written in Japanese**).

[31] Li Y, Zhou Y, Qiao W, et al. Application of decellularized vascular matrix in small-diameter vascular grafts. Front Bioeng Biotechnol. 2022;10:1081233. 36686240 10.3389/fbioe.2022.1081233 PMC9852870

[32] Katzman HE, Glickman MH, Schild AF, et al. Multicenter evaluation of bovine mesenteric vein bioprostheses for hemodialysis access in patients with an earlier failed prosthetic graft. J Am Coll Surg. 2005;201:223–30. 16038820 10.1016/j.jamcollsurg.2005.03.040

[33] Chemla ES, Morsy M. Randomized clinical trial comparing decellularized bovine ureter with expanded polytetra-fluoroethylene for vascular access. Br J Surg. 2009;96:34–9. 19108001 10.1002/bjs.6434

[34] Pashneh-Tala S, MacNeil S, Claeyssens F. The tissue-engineered vascular graft-past, present, and future. Tissue Eng Part B Rev. 2016;22:68–100. 10.1089/ten.teb.2015.0100. (**Epub 2015 Oct 8**). 26447530 10.1089/ten.teb.2015.0100 PMC4753638

[35] Negishi J, Hashimoto Y, Yamashita A, et al. Evaluation of small-diameter vascular grafts reconstructed from decellularized aorta sheets. J Biomed Mater Res A. 2017;105:1293–8. 28130834 10.1002/jbm.a.36017

[36] Mahara A, Somekawa S, Kobayashi N, et al. Tissue-engineered acellular small diameter long-bypass grafts with neointima-inducing activity. Biomaterials. 2015;58:54–62. 25941782 10.1016/j.biomaterials.2015.04.031

[37] Niklason LE, Gao J, Abbott WM, et al. Functional arteries grown in vitro. Science. 1990;284:489–93. 10.1126/science.284.5413.489 10205057

[38] Lawson JH, Glickman MH, Ilzecki M, et al. Bioengineered human acellular vessels for dialysis access in patients with end-stage renal disease: two phase 2 single-arm trials. Lancet. 2016;387:2026–34. 27203778 10.1016/S0140-6736(16)00557-2 PMC4915925

[39] Jakimowicz T, Przywara S, Turek J, et al. Five year outcomes in patients with end stage renal disease who received a bioengineered human acellular vessel for dialysis access. EJVES Vasc Forum. 2022;54:58–63. 35243473 10.1016/j.ejvsvf.2022.01.003 PMC8881722

[40] Wang J, Blalock SKF, Levitan GS, et al. Biological mechanisms of infection resistance in tissue engineered blood vessels compared to synthetic expanded polytetrafluoroethylene grafts. JVS Vasc Sci. 2023;4:100120. 10.1016/j.jvssci.2023.100120. (**eCollection 2023**). 37662589 10.1016/j.jvssci.2023.100120 PMC10474492

[41] Gutowski P, Gage SM, Guziewicz M, et al. Arterial reconstruction with human bioengineered acellular blood vessels in patients with peripheral arterial disease. J Vasc Surg. 2020;72:1247–58. 32093913 10.1016/j.jvs.2019.11.056

[42] Moore EE, Curi M, Namias N, et al. Bioengineered human arteries for the repair of vascular injuries. JAMA Surg. 2024;20:e244893. 10.1001/jamasurg.2024.4893. 10.1001/jamasurg.2024.4893 PMC11579887 39565635

[43] L’Heureux N, Dusserre N, Konig G, et al. Human tissue-engineered blood vessels for adult arterial revascularization. Nat Med. 2006;12:361–5. 16491087 10.1038/nm1364 PMC1513140

[44] McAllister TN, Maruszewski M, Garrido SA, et al. Effectiveness of haemodialysis access with an autologous tissue-engineered vascular graft: a multicenter cohort study. Lancet. 2009;373:1440–6. 19394535 10.1016/S0140-6736(09)60248-8

[45] Wystrychowski W, Garrido SA, Marini A, et al. Long-term results of autologous scaffold-free tissue-engineered vascular graft for hemodialysis access. J Vasc Access. 2024;25:254–64. 35773955 10.1177/11297298221095994

[46] Greco Song HH, Rumma RT, Ozaki CK, et al. Vascular tissue engineering: progress, challenges, and clinical promise. Cell Stem Cell. 2018;22:608. 10.1016/j.stem.2018.03.014. 29625073 10.1016/j.stem.2018.03.014 PMC5909377

[47] Wystrychowski W, McAllister TN, Zagalski K, et al. First human use of an allogeneic tissue-engineered vascular graft for hemodialysis access. J Vasc Surg. 2014;60:1353–7. 24103406 10.1016/j.jvs.2013.08.018

[48] L’Heureux N, McAllister TN, de la Fuente LM. Tissue-engineered blood vessel for adult arterial revascularization. N Engl J Med. 2007;357:1451–3. 10.1056/NEJMc071536. 17914054 10.1056/NEJMc071536

[49] Magnan L, Labrunie G, Fénelon M, et al. Human textiles: A cell-synthesized yarn as a truly “bio” material for tissue engineering applications. Acta Biomater. 2020;105:111–20. 31996332 10.1016/j.actbio.2020.01.037

[50] Borchiellini P, Rames A, Roubertie F, et al. Development and characterization of biological sutures made of cell-assembled extracellular matrix. Biofabrication. 2023;15:045018. 10.1088/1758-5090/acf1cf 37595608

[51] Sparks CH. Silicone mandril method of femoropopliteal artery bypass. Clinical experience and surgical technics. Am J Surg. 1972;124:244–9. 5045894 10.1016/0002-9610(72)90021-9

[52] Hallin RW, Sweetman WR. The Sparks’ mandril graft. A seven year follow-up of mandril grafts placed by Charles H. Sparks and his associates. Am J Surg. 1976;132:221–3. 10.1016/0002-9610(76)90051-9. 133619 10.1016/0002-9610(76)90051-9

[53] Nakayama Y, Ishibashi-Ueda H, Takamizawa K. In vivo tissue-engineered small-caliber arterial graft prosthesis consisting of autologous tissue (biotube). Cell Transpl. 2004;13:439–49. 10.3727/000000004783983828 15468686

[54] Nakayama Y, Kaneko Y, Okumura N, et al. Initial 3-year results of first human use of an in-body tissue-engineered autologous “Biotube” vascular graft for hemodialysis. J Vasc Access. 2020;21:110–5. 10.1177/1129729819852550. (**Epub 2019 Jum 6**). 31169047 10.1177/1129729819852550

[55] Kato N, Yamagishi M, Kanda K, et al. First successful clinical application of the in vivo tissue-engineered autologous vascular graft. Ann Thorac Surg. 2016;102:1387–90. 10.1016/j.athoracsur.2016.06.095. 27645948 10.1016/j.athoracsur.2016.06.095

[56] Higashita R, Miyazaki M, Oi M, et al. First-in-human results of an in-body tissue architecture-induced tissue-engineered vascular graft “Biotube” for application in distal bypass for chronic limb-threatening ischemia. J Vasc Surg Cases Innov Tech. 2022;8:488–93. 36052213 10.1016/j.jvscit.2022.07.007 PMC9424347

[57] Yamanami M, Kanda K, Morimoto K, et al. A tissue-engineered, decellularized, connective tissue membrane for allogeneic arterial patch implantation. Artif Organs. 2022;46:633–42. 10.1111/aor.14102. (**Epub 2021 Nov 12**). 34739732 10.1111/aor.14102 PMC9299228

[58] Yamanami M, Kanda K, Kawasaki T, et al. Development of xenogeneic decellularized biotubes for off-the-shelf applications. Artif Organs. 2019;43:773–9. 30697779 10.1111/aor.13432

[59] Murphy SV, Skardal A, Atala A. Evaluation of hydrogels for bio-printing applications. J Biomed Mater Res A. 2013;101:272–84. 22941807 10.1002/jbm.a.34326

[60] Borovjagin AV, Ogle B, Berry J, et al. From microscale devices to 3D printing: advances in the fabrication of 3D cardiovascular tissues. Circ Res. 2017;120:150–65. 28057791 10.1161/CIRCRESAHA.116.308538 PMC5224928

[61] Itoh M, Nakayama K, Noguchi R, et al. Scaffold-free tubular tissues created by a bio-3D printer undergo remodeling and endothelialization when implanted in rat aortae. PLoS One. 2015;10: e0136681. 26325298 10.1371/journal.pone.0136681 PMC4556622

[62] Itoh M, Mukae Y, Kitsuka T, et al. Development of an immunodeficient pig model allowing long-term accommodation of artificial human vascular tubes. Nat Commun. 2019;10:3625. 31113942 10.1038/s41467-019-10107-1 PMC6529409

[63] Itoh M. Scaffold-free autologous cell-based vascular graft for clinical application. In: Nakayama K, Moldovan NI, Sakamoto M, editors. Kenzan method for Scaffold-free biofabrication. Springer: Berlin; 2021. p. 117–25.

[64] Weinberg CB, Bell E. A blood vessel model constructed from collagen and cultured vascular cells. Science. 1986;231:398–400. 10.1126/science.2934816 2934816

[65] Matsuda T, Miwa H. A hybrid vascular model biomimicking the hierarchic structure of the arterial wall: neointimal stability and neoarterial regeneration process under arterial circulation. J Thorac Cardiovasc Surg. 1995;110:988–97. 7475165 10.1016/s0022-5223(05)80166-7

[66] Solan A, Mitchell S, Moses M, et al. Effect of pulse rate on collagen deposition in the tissue-engineered blood vessel. Tissue Eng. 2003;9:579–86. 13678437 10.1089/107632703768247287

[67] Luo J, Qin L, Zhao L, et al. Tissue-engineered vascular grafts with advanced mechanical strength from human iPSCs. Cell Stem Cell. 2020;26:251–61 (**e8**). 31956039 10.1016/j.stem.2019.12.012 PMC7021512

[68] Luo J, Qin L, Park J, et al. Readily available tissue-engineered vascular grafts derived from human induced pluripotent stem cells. Circ Res. 2022;130:9. 10.1161/CIRCRESAHA.121.320315 PMC9113663 35189711

